# Inferring Network Connectivity by Delayed Feedback Control

**DOI:** 10.1371/journal.pone.0024333

**Published:** 2011-09-28

**Authors:** Dongchuan Yu, Ulrich Parlitz

**Affiliations:** 1 Key Laboratory of Child Development and Learning Science of Ministry of Education, Southeast University, Nanjing, Jiangsu, China; 2 Research Center for Learning Science, Southeast University, Nanjing, Jiangsu, China; 3 Biomedical Physics Group, Max Planck Institute for Dynamics and Self-Organization, Göttingen, Germany; Humboldt University, Germany

## Abstract

We suggest a control based approach to topology estimation of networks with 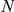 elements. This method first drives the network to steady states by a delayed feedback control; then performs structural perturbations for shifting the steady states 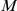 times; and finally infers the connection topology from the steady states' shifts by matrix inverse algorithm (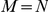) or 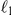-norm convex optimization strategy applicable to estimate the topology of sparse networks from 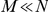 perturbations. We discuss as well some aspects important for applications, such as the topology reconstruction quality and error sources, advantages and disadvantages of the suggested method, and the influence of (control) perturbations, inhomegenity, sparsity, coupling functions, and measurement noise. Some examples of networks with Chua's oscillators are presented to illustrate the reliability of the suggested technique.

## Introduction

The research on complex networks [1]–[4] pervades almost all biological sciences, from gene network [5], [6] to system biology [7], from physiology [8]–[10] to psychology [11], to name just a few. Recent developments [12] in the quantitative analysis of complex networks, based largely on graph theory, have been rapidly translated to studies of brain networks. Mathematically, brain networks [12] can be described as graphs that are composed of nodes (vertices) denoting neural elements (neurons or brain regions) that are linked by edges representing physical connections (synapses or axonal projections) or functional ones based on imaging data. Current studies of brain networks focus on understanding the relation between network connectivity and function [12]. It turns out that small perturbations of structural and functional connectivity may dramatically change the function of networks and even lead to the occurrence of cognitive dysfunctions. In the context of brain functional networks based on imaging data [12], for example, one may quantify the functional connectivity between brain regions by analyzing the topological parameters (such as clustering coefficient, connectivity distribution, and average network distance) of the functional network, and the change of the topological properties has been considered as the pathophysiological mechanism of cognitive dysfunctions. In order to infer the emergent function of a real network, one first has to identify the underlying (functional and structural) connection topology.

Thus far a few methods have been developed for topology estimation using tools such as Pearson's correlation [13]–[15], phase synchronization [16], Bayesian estimation [17], [18], identical synchronization [19], perturbation [20]–[22], compressive-sensing [23], [24], direct reconstruction [25], [26], or linear state feedback control [27]–[29].

The Pearson's correlation method [13]–[15] is based on the following assumption: If the value of Pearson's correlation coefficient between two brain imaging time-series, representing the activities of two brain regions of interest, exceeds a threshold, then there exists a linkage between the two brain regions; otherwise, there is no connection between them. However, how to determine suitable thresholds is still an open problem and the assumption that correlation implies connections (or causality) is logically not sound [30], [31]. This problem also occurs with the phase synchronization approach [16] that depends on the following assumption: If the phase synchronization degree (or index) between two brain imaging time-series is above a threshold, then there exists a linkage between the two brain regions; otherwise, there is no connection between them. Again, the determination of the threshold remains a nontrivial problem. Furthermore, how to define the phase of complex systems still remains an open problem.

Bayesian estimation methods [17], [18] have been used to evaluate the connectivity between brain regions of interest with imaging data, but their efficiency and feasibility depend on the validity of the priors and the model adopted.

Network topology estimation using identical synchronization (which is conceptually equivalent to adaptive observer) was first developed in Ref. [19]. However, synchronization of networks may become an obstacle of topology estimation because synchronization leads to a situation where network connectivity information is hidden. Therefore, one has to complete the estimation processing as soon as possible (before the network is synchronous), otherwise one requires proper external perturbations to shift the network out of synchronous state.

Perturbation based method [20], [21] transforms the topology estimation problem into a matrix inversion task. It has been shown [21] that for sparsely connected networks, this matrix-inverse problem can be solved effectively using an 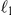-norm optimization strategy in combination with the well-known singular value decomposition technique. The perturbation method [20], [21], however, depends on the steady-state assumption (more precisely, it is assumed that the network to be analyzed always reaches a stable stationary state automatically) which is a restriction for some network systems with complex dynamical behaviors (including chaos). When the external perturbation matrix is unknown, a recursive strategy [22] can be used to estimate both the perturbation and connectivity matrices.

Some authors [23], [24] recently developed a so-called compressive sensing method that first formulates the dynamical system of interest as the following equation
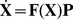
(1)with 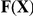 being a polynomial function and 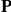 being parameter vector to be estimated, then obtains two data matrices 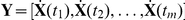 and 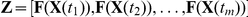, satisfying 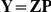, and finally estimate 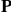 by an 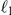-norm convex optimization processing. They showed that their method is effective to reconstruct dynamical systems [23] and network topology [24]. However, such a method requires a differential estimator that may be sensitive to measurement noise. Furthermore, complex dynamical systems usually cannot be described by Eq. (1), more precisely, their dynamical equation in general is non-polynomial and does not linearly depend on the parameters. It should be remarked that the performance of 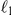-norm convex optimization strategy usually becomes bad when the sparsity of networks decreases, as will be shown below (cf. Fig. 8).

Timme's recent work [26] analyzed the possibility of direct topology reconstruction from dynamical trajectories. Remarkably, the question how the parameters (e.g. sampling rate, observation time, and external noise) influence the performance of topology reconstruction is discussed in detail. The reliability of his method [26] has been demonstrated clearly. As a minor drawback, his method requires some prior knowledge about local dynamics of each node, and a differential estimator that may be sensitive to measurement noise.

To use the perturbation method also for networks with complex dynamics, a linear state feedback control based method [27]–[29] was suggested very recently, and can be used to estimate topology by exploiting information obtained from the observed steady-state responses of each node. However this method has some limitations. For instance, one generally has to assume that all state variables of each node are completely measurable and all state components of each node admit an external input. Furthermore, a high-gain feedback control will be involved in some cases.

In brief, most of developed topology estimation methods have their advantages and disadvantages, and thus far the topology estimation issue remains an open problem. Here we make an effort to remove some drawbacks of previous methods, and show that the connection topology of complex dynamical networks can be identified by exploiting information obtained from shifted steady states that are stabilized by means of *multiple delay feedback control* (MDFC) [32]. This control approach is combined with some methods [21] for detecting connectivity of networks under the assumption that a stable stationary state exists (also called *steady state assumption*). However, in contrast to that work, our topology detection method is applicable to dynamical networks with complex dynamical behaviors (far from stationarity) and does not depend on the steady state assumption. Furthermore, our method is possible to be applied in a challenging scenario where only one state variables of each node are measurable and accessible, and does require only a little structure information about the networks under study.

## Results

### Theory

We consider a network of interacting dynamical systems, given by
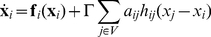
(2)where 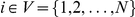; 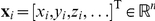 is the state vector of the 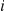
th element (or node); 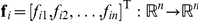 describes the dynamics of the 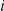
th element. For simplicity we assume that only the first components of each element are connected to each other (a more general case will be treated elsewhere). Here 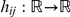 is a coupling function and 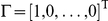. The topology of the network connections is determined by the adjacency matrix 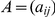: 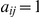 if there exists a connection from the 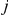
th node to the 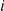
th node; and 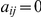 otherwise. We shall show that MDFC [32] is very efficient to shift the steady states and the steady states' shifts enable us to uncover the connection topology in terms of an estimation of the elements of the matrix 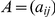.

We restrict ourselves to the case that only the coupling variables, namely 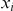, can be measured (or monitored) and we add the control term
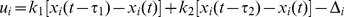
(3)to only the first equation of each element, where delay times 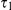 and 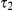 and control gains 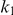 and 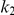 are *uniform* for all elements. For 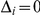, the control signal (3) becomes the original MDFC [32]. Here we shall *first* use distinct 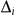 for each element to shift the steady states which are stabilized by MDFC and *then* show that the steady states' shifts enable us to uncover the connection topology.

#### Steady-state stabilization

The network system (2) under the control signal (3) can then be rewritten in a compact form:
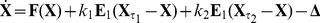
(4)where 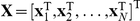, 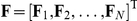, 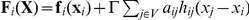, 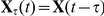, 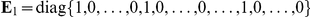, and 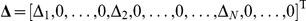.

**Assumption 1**: For function 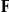, there exists constants 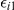 and 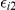 (depending on the property of function 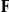) such that the equation 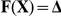 has at least one real solution 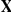 for any 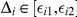 for all 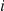.

If system (2) without any perturbation has at least one equilibrium, which usually is satisfied for most of networks, then equation 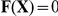 has at least one real root. By using the continuity of function 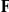 (because 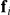 is continuous for all 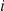), it follows that when constants 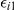 and 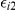 are close to zero, equation 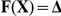 has at least one real root. This indicates that Assumption 1 is not really a restriction in practice.

The following theorem is the foundation of topology identification and provides conditions under which the system (4) is locally asymptotical stable at a stationary state. Detailed discussion about Theorem 1 can be found in **Discussion** Part.

**Theorem 1**: The system (4) (with 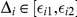 for all 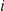) is locally asymptotical stable at a stationary state 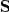, satisfying
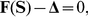
(5)provided that: (i) Assumption 1 holds; and (ii) all roots of the characteristics equation 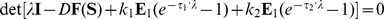 have negative real parts, where 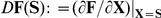 is the Jacobian matrix.

*Proof*: The existence of 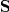 satisfying Eq. (5) is straightforward if Assumption 1 holds. Now we analyze the stability of the stationary state 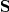.

Let 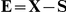. Then we can conclude from Eq. (4) that
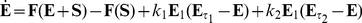
(6)where Eq. (5) has been used.

Locally linearizing the above system around the origin results in
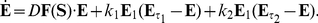
(7)


Therefore, in terms of the standard linear system theory, the stability of the error system (7) determines by the characteristics equation 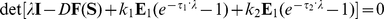. If all roots of the characteristics equation have negative real parts, then the asymptotic stability of the error system (7) is satisfied. This completes the proof.

#### Steady-state shifts

If proper 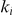, 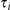, and 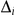 are chosen such that Theorem 1 is fulfilled (see **Discussion** Part for further information), then one can stabilize the steady state 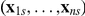, satisfying 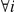


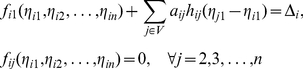
(8)where 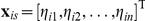 is the steady state of the 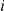
th element.

If 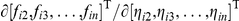 is nonsingular, then one can conclude from the implicit function theory [33] that there exists a mapping 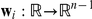 such that
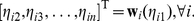
(9)


Substituted into the first equation of Eq. (8) this yields
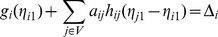
(10)where 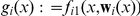.

As will be shown below, Eq. (10) is the foundation of the topology estimation method to be suggested, and has reduced the original 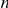-dimensional problem to an 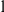-dimensional (1D) one. It should be remarked that Eq. (10) is satisfied, provided that (i) Equation (8) has at least one real solution; (ii) the steady state satisfying Eq. (8) can be stabilized by MDFC; and (iii) 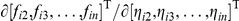 is nonsingular.

We now show that shifting the steady states of the network system 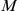 times by 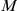 structural perturbations enables us to uncover the network connectivity (where 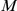 depends on the network size 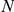).

For the 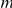
th perturbation, we replace the control constant 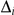 by 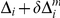 for each node 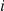 such that the steady states of the coupling variables are shifted from 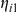 to 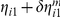 for all 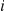. Then the resulting steady state response equations of the coupling variables read
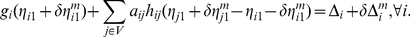
(11)


For sufficiently small perturbations 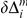, we approximate 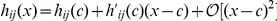 and 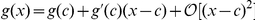. Subtracting Eq. (11) from Eq. (10), we then obtain
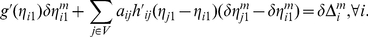
(12)


Let 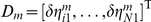, 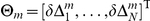, and 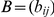 with
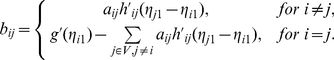
(13)


Then the set of equations (12) can be rewritten in a compact form
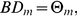
(14)which contains 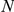 equations that restrict the 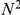 elements 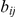.

Perturbing the steady state of the network system 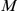 times, we achieve
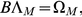
(15)where 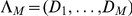 and 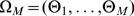.

To summarize the above analysis, Eq. (15) is fulfilled if and only if: (i) Equation (8) has at least one real solution; (ii) the steady states satisfying Eqs. (10) and (11) can be stabilized by MDFC; (iii) 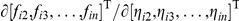 is nonsingular; and (iv) perturbations 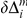 are sufficiently small for all 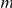.

#### Topology estimation using matrix inverse algorithm

Equation (15) actually contains 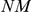 conditions that restrict the 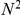 elements 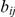. Hence, after performing 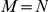 perturbations, all elements 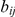 can be estimated by 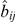, given by
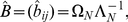
(16)if the inverse of 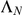 exists.

It follows that if all elements 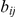 can be estimated with high accuracy (more precisely, there exists a sufficiently small 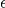 such that 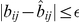) , then all off-diagonal elements 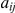 can be identified from Eq.(13): 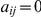 when 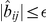; and 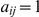 otherwise. In practice, one may follow the SDTIA algorithm [28] and divide all values 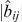 into two sets: 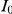 containing all elements 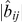 corresponding to 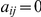 and 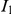 containing all elements 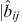 corresponding to 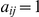, by the following steps:

**Step 1.** Calculate elements 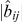 for all 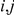.
**Step 2.** Order (or arrange) all elements 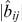 in an ascending sequence and obtain a new sequence 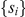.
**Step 3.** The critical point sequence number 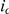 of set 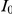 is determined by the rule: 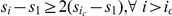.

**Figure 1:**
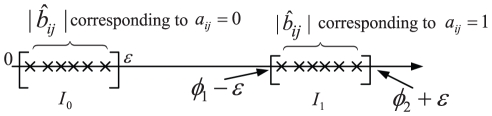
The condition to ensure a successful topology reconstruction using the SDTIA algorithm [**28**]. Sets 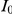 and 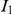 contain all elements 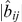 corresponding to 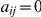 and that corresponding to 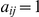, respectively, where 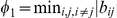 and 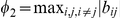.

As clearly shown in Fig. 1 that when 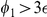 with 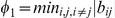 and 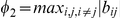, the distance between sets 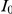 and 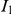 is larger than the length of set 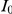, and thereby one can distinguish the sets 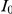 and 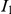 by the above steps (SDTIA algorithm [28]) and reconstruct the network topology in terms of an estimation of all elements of the matrix 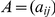, where the distance between two point sets is equal to the minimal distance between any two points which are taken from different sets, and the length of a point set is the difference between the maximal and minimal values in the set. Therefore, the smaller the value of 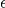 and the bigger the value of 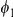, the higher the possibility of successful topology reconstruction.

#### Topology estimation using 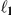-norm optimization strategy

Topology estimation using Eq. (16) requires 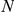 perturbations and becomes “costly” and less effective when the network size 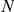 is very large. However, for sparsely connected networks, it turns out that by using a 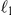-norm convex optimization strategy to be shown below, we can accurately and efficiently approximate all elements 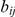 from Eq. (15) with 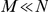.

We transpose Eq. (15) and rewrite it as
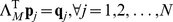
(17)where 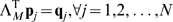 and 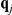 are the 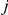
th column vector of matrices 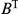 and 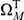, respectively.

The estimated value of each 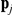, referred to as 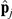, can be determined by solving the following convex optimization problem
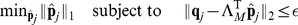
(18)where 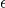 is the tolerance (in the following simulations, 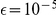), 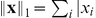 is the 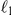-norm of vector 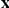 and 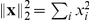.

The advantage of choosing the formulation (18) is that one can determine the network with a minimal number of connections (each vector 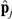 will have a minimal number of nonzero elements) and it can be solved in polynomial time with some standard scientific softwares (e.g., Matlab toolbox CVX Ver1.1 [34]). By this 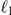-norm convex optimization strategy, we can determine the matrix with minimal driving connections for each node; hence we can effectively estimate all 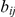 for sparsely connected networks when 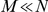 perturbations are performed, as will be illustrated below. Again, one may follow the SDTIA algorithm [28] (shown above) for an effective topology reconstruction.

#### Topology estimation quality

Following Timme's work [26], we define the normalized estimation error 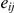 of each element 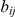 by
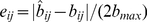
(19)where 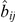 is an estimation of 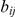, and 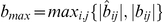.

We further define the estimation accuracy 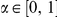 such that 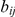 can be identified correctly if
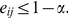
(20)This implies from Fig. 1 that 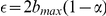 and thereby the topology can be estimated correctly if
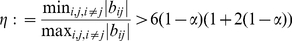
(21)where 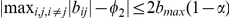 and 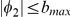 are used. Therefore, the bigger the values of 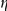 and 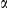, the higher the topology estimation accuracy. Based on the condition (21), the minimal value of 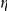 being supported for a successful topology reconstruction is determined by the maximal value of 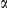 satisfying the condition (20).

The estimation accuracy of 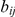 is crucial for topology reconstruction, so it is of importance to quantify the estimation quality of values 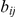. Here we qualify the estimation accuracy of all non-diagonal elements 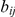 as a whole by the variable 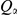, given by
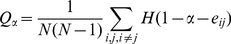
(22)where 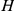 is the Herviside step function, i.e., 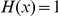 for 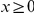 and 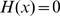 otherwise. This definition is a little bit different from Timme's work [26] that considered the estimation of all elements 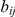. It is clear that the bigger the values of 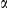 and 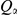, the higher the estimation accuracy of all non-diagonal elements 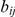. Based on this observation, we restrict ourselves and assume that an effective network topology reconstruction is said to occur when 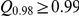.

### Simulation

**Figure 2:**
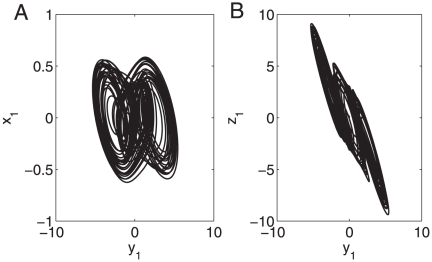
Chaotic behavior of system (23) with 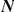 = 16 and node-pair connection probability 0.3. (A) 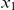-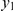 phase figure. (B) 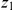-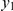 phase figure.

To illustrate the above topology estimation methods, we use a network of coupled Chua's circuits, given by
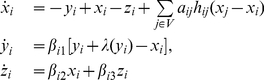
(23)where 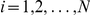, 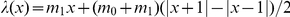, and parameters 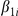, 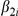, and 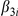 are uniformly distributed in ranges [35.6, 35.75], [75.6, 75.75], and [1.103, 1.253], respectively. Furthermore, 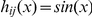 are for any 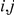, so coupling functions 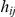 do not contain any information about the network topology (some further discussion about coupling functions can be found in **Discussion** Part). In this case, system (23) may display complex dynamical behavior (including chaos), as illustrated in Fig. 2. In the following, we first illustrate the steady state stabilization and shifts numerically. Then, based on steady state shifts and measurement, we show two methods for topology estimation, i.e., matrix inverse and 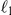-norm convex optimization strategy, with estimation accuracy being quantified by 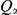.

**Figure 3:**
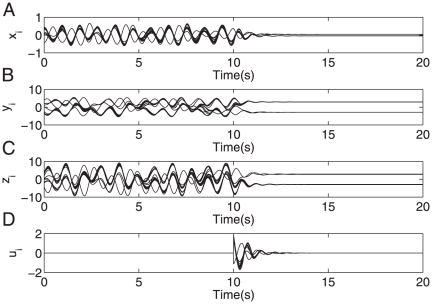
Stable stabilization of system (23). (A)–(C) present the dynamic behavior of system (23) (with 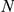 = 16 and node-pair connection probability 0.3) being driven for 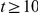 by the control signal (3) shown in (D) (with 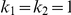, 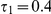, 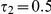, 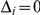 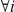).

**Figure 4:**
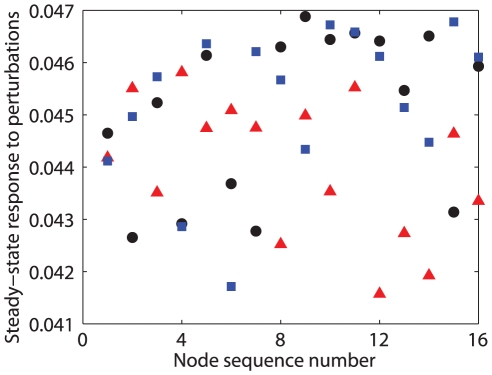
Steady state shifts. Black circles plot the steady state response of system (23) with 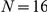 by control signal with 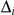 being randomly chosen from the range 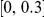. Blue squares and red triangles represent the steady state response to two random perturbations 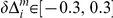 on the values 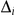, respectively. All plots show only the steady state response of the first state of each Chua oscillator.

Following Ref. [32], we can determine suitable control parameter values 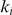 and 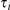 by a search strategy. We numerically found that there is a big window for the control parameters 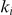 and 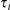 such that system (2) can be driven to a steady state by the MDFC (3), as illustrated in Fig. 3 as a typical example. It is clear from Fig. 3D that MDFC is very effective for steady state stabilization. Furthermore, when MDFCs with distinct 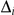 are used, the steady-state response shift phenomenon can be observed (cf. Fig. 4 for a representative result). Those steady state shifts are the foundation of topology estimation, as shown above (cf. **Theory** Part).

When system (23) is driven to a steady state 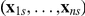 with 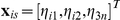 being the steady state of the 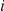
th element, then one can easily confirm that
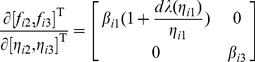
(24)is nonsingular and
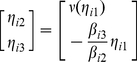
(25)where 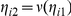 is the unique solution of equation 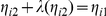.

Therefore, Eq. (10) is fulfilled. This implies that shifting and measuring the steady state response of the first state of each node becomes possible for a successful topology reconstruction. In the following, we show two methods for topology estimation, i.e., matrix inverse and 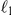-norm convex optimization strategy.

**Figure 5:**
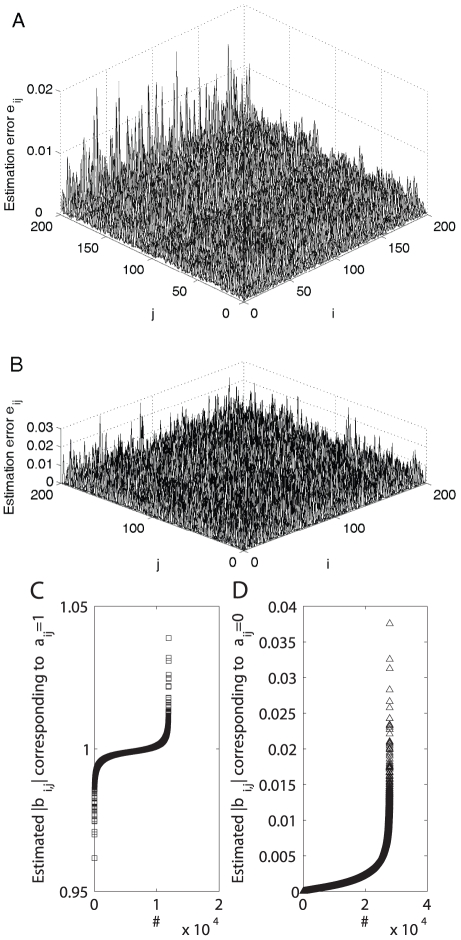
Topology estimation: Matrix inverse algorithm VS 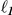-norm optimization strategy. The estimation error surfaces are calculated using two methods for a undirected network (23) with 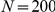 and node-pair connection probability 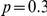: (A) matrix inverse algorithm (with 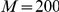, 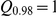); and (B) 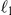-norm convex optimization strategy (with 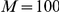, 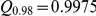), respectively. With the normalized error 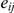 shown in Panel (A), Panels (C)–(D) plot the estimated 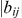 corresponding to 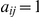 and that corresponding to 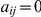 after being sorted with ascending order, respectively. It is clear from Panels (C)–(D) that one may identify all parameters 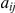 correctly by the SDTIA algorithm [28].

As a representative result using the matrix inverse algorithm (16) for topology estimation, Fig. 5A shows the estimation error 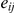 of elements 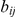 for a random directed network of interacting Chua's oscillators. It is clear that all elements 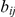 have been reconstructed effectively with 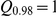 (due to 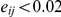 for all 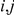). With this high (normalized) estimation accuracy of 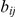, one may identify all parameters 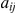 correctly by the SDTIA algorithm [28] (also shown above), as illustrated in Figs. 5C–5D where the estimated 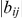 (with □) corresponding to 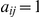 are bigger than that (with 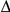) corresponding to 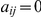.

**Figure 6:**
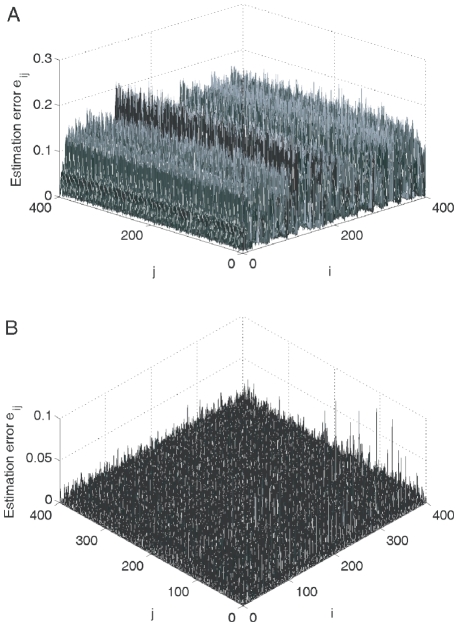
Topology estimation: Matrix inverse algorithm VS 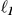-norm optimization strategy. The estimation error surfaces are calculated using two methods for a undirected network (23) with 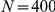 and node-pair connection probability 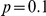: (A) matrix inverse algorithm (with 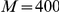, 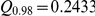); and (B) 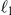-norm convex optimization strategy (with 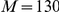, 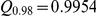), respectively.

The matrix inverse method for topology reconstruction requires 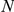 perturbations and becomes “costly” and less effective when the network size 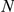 is very large. However, such a drawback for sparsely connected networks may be relaxed by the 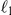-norm convex optimization strategy described in Eq. (18). As typical examples, Fig. 5B and Fig. 6B shows that an acceptable topology estimation accuracy (i.e., 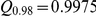, 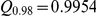) can be obtained when only 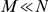 perturbations are performed.

Furthermore, the matrix inverse method may lead to wrong conclusion in some cases due to the ill-condition problem of the matrix inverse, as illustrated in Fig. 6A where 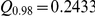, implying a bad estimation result, is achieved. However, for sparse networks, such a drawback may be removed by the 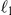-norm convex optimization strategy, as shown in Fig. 6B where 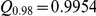.

**Figure 7:**
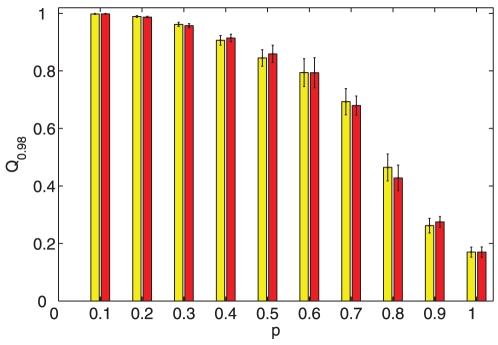
The influence of node-pair connection possibility on topology reconstruction of random networks. The estimation error 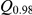 changes with the node-pair connection possibility 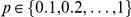 for two cases, i.e., undirected (yellow bars) and directed (red bars) networks. There, 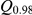 is calculated using the 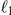-norm convex optimization strategy (with 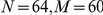). Furthermore, each bar represents the result of averaging over 30 random perturbations (with 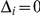 and 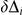 being uniformly distributed in the range 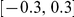) and the standard square error is given as well.

**Figure 8:**
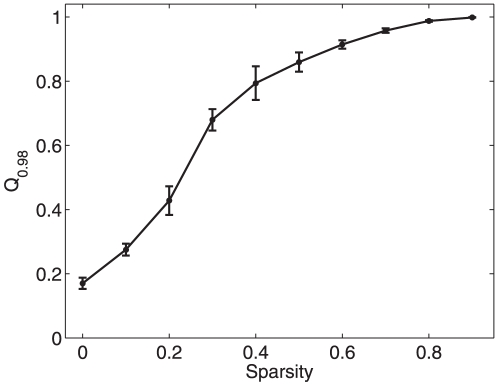
The influence of the sparsity on topology reconstruction. The estimation error 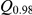 changes with the sparsity of directed random networks. There, the sparsity is defined as the ratio of the number of zero non-diagonal elements 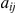 to 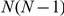, and 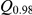 is calculated using the 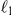-norm convex optimization strategy (with 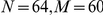). Furthermore, each black point represents the result of averaging over 30 random perturbations (with 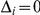 and 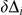 being uniformly distributed in the range 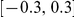) and the standard square error is given as well.

The question how to choose control parameters becomes crucial for steady state shifts which are the foundation for topology reconstruction. For simplicity, in the above simulation, we let 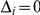 and choose parameters 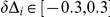 randomly. We now analyze the influence of perturbation parameters 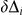 on topology estimation. Figure 7 summarizes our results and shows that the estimation accuracy 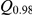 using the 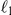-norm convex optimization strategy changes with the node-pair connection possibility 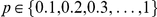 for two cases, i.e, undirected (cf. yellow bars) and directed (cf. red bars) networks. There, each bar represents the result of averaging over 30 random perturbations (with 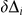 being uniformly distributed in the range 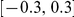) and the standard square error is given as well. From Fig. 7 we may draw the following conclusions: (i) the performance of topology reconstruction using the 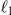-norm optimization strategy becomes bad when 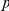 increases; (ii) The estimation accuracy 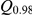 is not sensitive to the choice of perturbation parameters 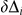 when 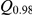 is close to one; (iii) There is no distinct difference between undirected (cf. yellow bars) and directed (cf. red bars) networks. This indicates that the performance of topology reconstruction using the 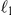-norm convex optimization strategy is not sensitive to the inhomegeneity but sparsity (cf. Fig. 8).

**Figure 9:**
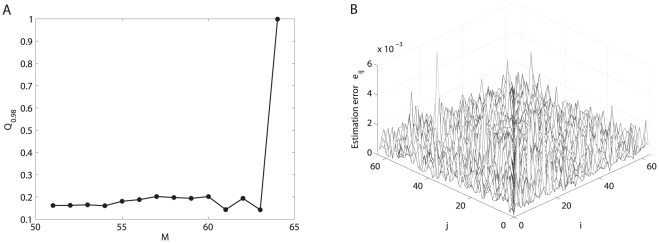
Topology reconstruction of full-connected networks with 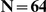. (A) The estimation error 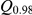 changes with the number 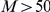 of perturbations, where 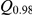 is calculated using the 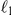-norm convex optimization strategy. (B) The estimation error surface (with 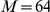, 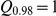) is calculated using the matrix inverse algorithm for the same system shown in Panel (A).

Note that the 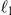-norm convex optimization strategy is very effective for sparsely connected networks only. Hence, for non-sparsely connected networks, this optimization method usually has to require that almost all nodes are perturbed, as illustrated in Fig. 9A as a representative result. In this case, the 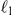-norm convex optimization strategy has no any clear advantage compared to the matrix inverse algorithm (cf. Fig. 9B) that uses an 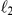-norm optimization processing.

**Figure 10:**
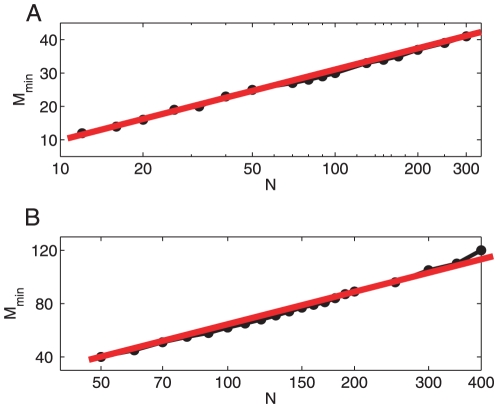
The functional relation between 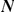 and 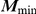. The logarithmic-linear plot of the relation of 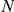 and 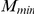 for two cases: (A) 4-nearest-neighbor coupled network; and (B) directed network of nodes randomly connected with possibility 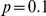. There, the best logarithmic fitting are plotted with red lines for both cases.

As mentioned above, we restrict ourselves and assume that an effective network topology reconstruction is said to occur when 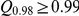. Based on this rule, we now analyze numerically the relation between the minimal number of perturbations, referred to as 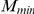, that are required for a successful topology reconstruction satisfying 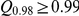, and the network size 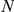. Figure 10 summarizes our results and shows the logarithmic-linear plot of the relation of 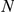 and 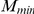 for two cases, i.e., 4-nearest-neighbor coupled network and directed network of nodes randomly connected with possibility 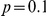. There is a clear logarithmic-linear relation between 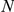 and 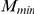. This result is consistent with Timme's work [21], and implies that we need less control applications (perturbations) than the size of the networks under study.

**Figure 11:**
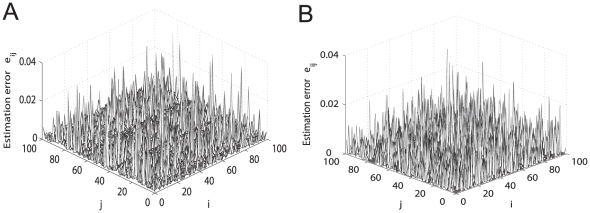
The influence of measurement noise on topology reconstruction. The estimation error surfaces of a directed network (23) with 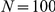 and node-pair connection probability 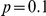 are calculated using 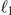-norm convex optimization strategy for two cases: (A) the presence of 5% measurement noise (with 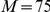 and 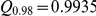); and (B) the absence of measurement noise (with 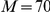 and 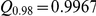).

Measurement noise cannot be avoided in some cases and usually deteriorates the control performance of high-gain control methods because measurement noise is largely amplified. Fortunately, the MDFC method does not belong to high-gain control [28] and can stabilize stationary states with very small gain (indeed 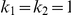 was used in all simulation results in this paper). This implies that our topology estimation method is applicable to network systems in the presence of measurement noise, as illustrated in Fig. 11A where results are shown obtained from observed signals contaminated with 5% measurement noise. We found that more perturbations are generally required in the presence of measurement noise (cf. Fig. 11A where 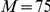, 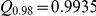) compared to the case in the absence of measurement noise (cf. Fig. 11B where 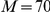, 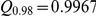).

**Figure 12:**
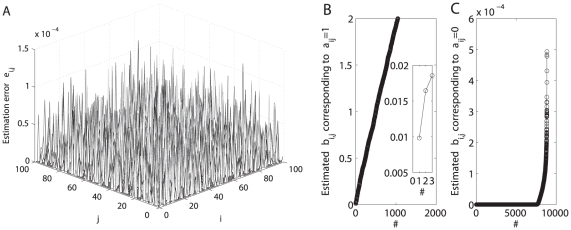
Topology reconstruction in the case of 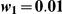, 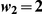, and 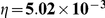. (A) The estimation error surface of a directed network with 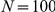 and node-pair connection probability 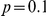 is calculated using 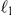-norm convex optimization strategy with 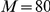. With the normalized estimation errors 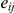 shown in Panel (A), Panels (B)–(C) plot the estimated 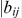 corresponding to 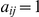 and that corresponding to 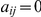 after being sorted with ascending order, respectively. Insert in Panel (B) shows a local augment. It is clear that the minimal value of estimated 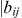 shown in Panel (b) is more than twice the maximal value of estimated 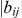 shown in Panel (C), and thereby one may identify all parameters 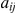 correctly by the SDTIA algorithm [28].

**Figure 13:**
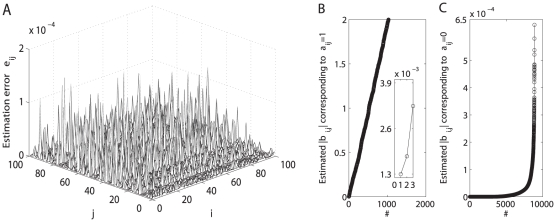
Topology reconstruction in the case of 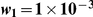, 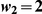, and 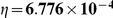. (A) The estimation error surface of a directed network with 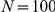 and node-pair connection probability 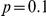 is calculated using 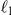-norm convex optimization strategy with 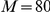. With the normalized estimation errors 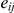 shown in Panel (A), Panels (B)–(C) plot the estimated 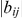 corresponding to 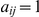 and that corresponding to 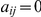 after being sorted with ascending order, respectively. Insert in Panel (b) shows a local augment. It is clear that the minimal value of estimated 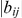 shown in Panel (b) is more than twice the maximal value of estimated 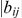 shown in Panel (c), and thereby one may identify all parameters 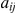 correctly by the SDTIA algorithm [28].

Finally, we analyze the influence of 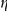 on topology estimation, and revisit the network (23) but assume 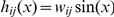 with 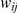 being uniformly distributed in range 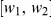 such that the value of 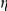 can be changed with the choice of parameters 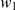 and 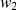. Figures 12 and 13 summarize our results and show in both cases (i.e., 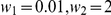 and 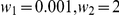) that the minimal value of estimated 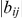 corresponding to 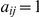 is more than twice the maximal value of that corresponding to 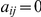, and thereby one may identify all parameters 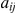 correctly by the SDTIA algorithm [28]. Furthermore, the ratio of the distance between sets 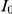 and 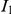 to the maximal value of set 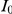 roughly increases with the value of 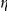 where the definition of sets 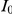 and 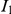 is illustrated in Fig. 1. Therefore, there exists a critical value 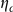 such that if 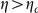 is fulfilled, then one may identify all elements 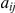 correctly. On the other hand, when 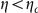, the boundary between sets 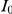 and 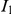 will become unclear and some elements 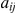 cannot be identified correctly. Even under such a circumstance, it is still possible to estimate partial elements 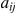 correctly if a suitable strategy is used to delete those elements 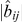 contaminating the boundary between sets 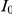 and 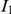. Detailed analysis is now under our investigation and will be reported elsewhere.

## Discussion

### Delayed feedback control

It has been shown experimentally [35]–[38] that Pyragas's delayed feedback control method [39], which feeds the amplified difference of a monitor (or measurable) variable and its delayed component back to the controlled system, is applicable and very effective to stabilize unstable period orbits as well as unstable equilibrium points. Some advantages of Pyragas's delayed feedback control method include: (i) it feeds the amplified difference of a monitor (or measurable) variable and its delayed component back to the controlled system but does not use any structure information about the controlled system; (ii) it is noninvasive, that is, the control signal approaches to zero after a unstable period orbit or a unstable equilibrium point is stabilized; and (iii) it can easily be realized using analog or digital devices. Some extended versions using more delayed components have also been developed for improving further the control performance, such as extended time delay auto synchronization [40], [41] and multiple delay feedback control [32], [42], [43] methods.

Delayed feedback control methods [32], [35]–[43] are very efficient for stabilizing unstable periodic orbits or unstable stationary states in various real systems such as optics, semiconductors, networks of chemical oscillators, and reaction-diffusion systems. Our previous work [32], [42], [43] showed that the performance of stabilizing stationary states is significantly improved using several independent delay times.

**Although the reliability of all delayed feedback control methods for stabilizing unstable period orbits and unstable equilibrium points has been illustrated by various experiments, the theoretical analysis and mechanism of delayed feedback control is still far from strictness and completeness** [**44]**–[47****]. Fortunately, we found that the steady state stabilization based on MDFC is always possible for a large class of dynamical networks. In practice, one can usually determine suitable control parameter values by a search strategy, as illustrated in previous work [32].

Thus far the research on delayed feedback control focused on stabilizing unstable period orbits and unstable equilibrium points of chaotic systems. In this paper, we show a potential application of using delayed feedback control for topology reconstruction. Compared to previous linear state feedback control method [27]–[29] which in general requires high-gain control and full state feedback (i.e., all state components of each node are measurable and accessible), the suggested delayed feedback control method is applicable even in a challenging scenario where only one state variables of each node are measurable and accessible.

### Extension to more general coupling functions

Our method can also be extended to networks with more general coupling functions but does not limit to those with only the state-difference form 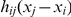. To demonstrate this point more clearly, we consider the following network
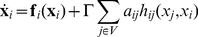
(26)where all variables follow the same definition in system (2) except the coupling functions 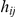. Here 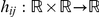. Again, we assume that system (26) can be driven to a steady state by the control signal (3). In this case, following similar steps developed for the state-difference form, one can easily see that Eq. (12) now reads
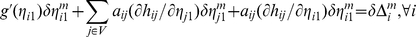
(27)where the first order approximation 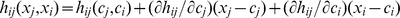 is used.

This implies that Eq. (14) is again fulfilled but the matrix 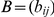 now reads
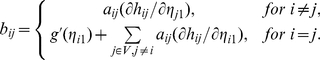
(28)


**Figure 14:**
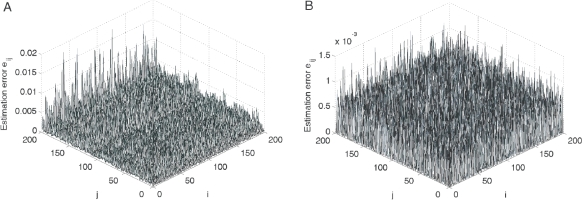
Topology estimation of network (26) with 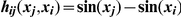. The topology estimation error surfaces are calculated using two methods for a undirected network with 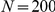 and node-pair connection probability 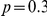: (A) matrix inverse algorithm (with 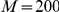, 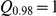); and (B) 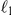-norm optimization strategy (with 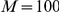, 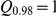), respectively.

Therefore, our methods using matrix inverse algorithm and 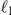-norm convex optimization strategy can be extended to topology reconstruction of network (26) with more general coupling form, as illustrated in Fig. 14 where 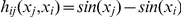 and the network topology can be estimated effectively.

### Implementation and error sources

We briefly outline our method for topology estimation:

- Drive the network (with 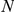 nodes) to a steady state by control signal (3) with 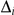 (usually 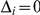), and measure the resulting steady state response 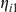 for all 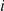;
- Perturb the control signal (3) (i.e., replace 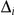 by 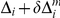 where 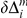 is randomly chosen from the range [-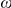, 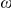]) 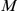 times, and measure the resulting steady state response 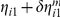 for all 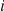;
- Estimate all non-diagonal elements 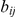 using the matrix inverse algorithm (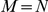) or the 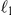-norm convex optimization strategy (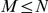);
- Infer all non-diagonal elements 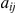 from estimated 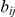 by the SDTIA algorithm [28].

One may see from the above steps that the topology estimation error may come from different sources: (i) Steady state control; (ii) Steady state measurement; (iii) The first order approximation concerning functions 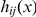; (iv) The matrix inverse operation error (for the matrix inverse algorithm) or the optimization error (for the 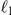-norm convex optimization strategy); and (v) The value of 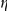.

As described above, delayed feedback control methods [35]–[38] are very efficient for stabilizing stationary states in various real systems such as optics, semiconductors, networks of chemical oscillators, and reaction-diffusion systems. Therefore, steady state control usually cannot be considered as an error source, as illustrated in Figs. 5, 6B, 9B, 10, 11, and 12.

Measurement of steady states also cannot be taken as a major error source, as illustrated in Fig. 11 where acceptable results are shown obtained from observed signals contaminated with 5% measurement noise.

It is clear that if sufficiently small perturbations 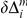 are used, then the first order approximation of functions 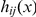 is reasonable and cannot be taken a major error source of topology estimation. This point has been supported by many numerical examples (cf. Figs. Figs. 5, 6B, 9B, 10, 11, and 12).

For the matrix inverse algorithm, a major error source may come from the inverse operation itself, as illustrated in Fig. 6A where a bad estimation result (with 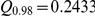) is achieved due to the ill-condition problem of the matrix inverse operation.

For the 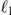-norm convex optimization strategy, a major error source may come from the sparsity of networks under study, as illustrated in Fig. 8. This is consistent with the fact that the 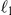-norm convex optimization strategy is effective for sparsely connected networks only.

The influence of 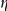 on topology estimation has been illustrated in Figs. 12–13. It is clear that the ratio of the distance between sets 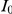 and 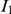 to the maximal value of set 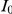 roughly increases with the value of 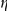 where the definition of sets 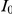 and 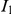 is illustrated in Fig. 1. Therefore, there exists a critical value 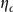 such that if 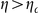 is fulfilled, then one may identify all elements 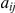 correctly. It should be remarked that the value of 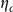 is determined by the control signal (3), the coupling functions, the equilibria of network (2), and the initial states. If the network under study has more than one equilibrium, then it is still possible to change the value of 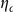 by choosing the proper time to perform the steady state control to shift the equilibrium of the network dramatically. However, such a strategy in principle has to require some prior knowledge about the the equilibria of the network, and thereby has its restriction in some applications.

### Advantages and disadvantages of our method

Some advantages of our method include:

- If network synchronization occurs and leads to vanishing coupling terms, then the network connectivity information is hidden and cannot be recovered with time-series analysis methods [19], [24], [26]. However, our topology reconstruction method is applicable to synchronous networks;
- Previous topology reconstruction method [27], [28] based on steady-state stabilization generally has to assume that all state variables of each node are completely measurable and all state components of each node admit an external input. However, our method is applicable even in a challenging scenario where only one state variables of each node are measurable and accessible;
- Our method requires only small control injection and does not belong to a kind of high-gain control [27], [28]. Hence it is not sensitive to measurement noise and may achieve better performance than high-gain control method [27], [28] and the methods using differential estimator in the presence of measurement noise;
- Previous time-series methods [19], [26] require a lot of information about the local dynamics of each node and coupling functions. This is really a restriction in some applications. However, our method does require only a little structure information about the controlled networks, and provides a promising solution for topology reconstruction if the required control perturbations are allowed.

On the other hand, our method also possesses some disadvantages:

- Our method is applicable to topology estimation of sparsely connected networks with size 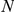 when 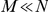 perturbations are performed, but in general one has to measure the steady state response of all nodes and the measurement “cost” increases linearly with the size of networks, even when only partial connections of interest require to be estimated. Such a drawback also exists for most of previous methods except the high-gain control method [28];
- Steady state stabilization and shifts are the foundation of our method. However, such a kind of steady state control (or perturbation) will influence the dynamical behavior of systems, so our method may fail for systems that do not support the required steady state control. In this case, previous time-series methods [19], [24], [26] might be considered as a potential strategy for topology reconstruction.
- Our method may in principle fail when time-varying topology is required to be reconstructed. In such a circumstance, previous time-series methods [19], [24], [26] might be applicable for correct estimation.

### Potential applications

Previous works have shown the importance of topology connections on spatiotemporal pattern of networks of coupled chemical oscillators [48]–[51]. Furthermore, delayed feedback control has effectively been applied to stabilize (unstable) steady states of chemical oscillators (cf. Ref. [52] for a representative result). Therefore, our method is possible to be used to reconstruct the connection topology of interacting chemical oscillators. Another possible application is to reconstruct topology of gene networks [22] by delayed feedback control, provided online measurement and injection techniques are feasible. Generally, the suggested technique enables us to identify the connection topology of real networks (including circuit networks and interacting coupled chemical oscillators [48]–[51]) which allow the required control applications (perturbations). Some possible experimental research is now under our investigation.
